# Defining Clinically Meaningful Thresholds for 12-Month Patient-Reported Outcomes in Total Hip Arthroplasty; Toward Improving Threshold Accuracy

**DOI:** 10.1016/j.artd.2025.101649

**Published:** 2025-03-03

**Authors:** Julia E.J.W. Geilen, Thomay-Claire A. Hoelen, Martijn G.M. Schotanus, Wouter L.W. van Hemert, Anneke Spekenbrink-Spooren, Bert Boonen, Jasper Most

**Affiliations:** a Department Orthopedics and Traumatology, Zuyderland Medical Center, Sittard-Geleen, The Netherlands; b Department Orthopedic Surgery, CAPHRI, Maastricht University Medical Center+, Maastricht, The Netherlands; c Dutch Arthroplasty Register (LROI), 's-Hertogenbosch, The Netherlands; d Department Epidemiology, CAPHRI, Maastricht University Medical Center+, Maastricht, The Netherlands

**Keywords:** Minimal clinically important change (MCIC), Minimal clinically important differences (MCIDs), Patient-acceptable symptom state (PASS), Total hip arthroplasty (THA), Patient-reported outcome measures (PROMs), Function

## Abstract

**Background:**

Clinically meaningful thresholds for patient-reported outcomes are relevant to define and predict success of total hip arthroplasties (THAs). Defining and offering thresholds must consider preoperative symptom severity.

**Methods:**

In this retrospective study of 40,213 primary total hip replacements registered in the Dutch Arthroplasty Register (2016-2018), receiver operating curve analysis was used to define minimal clinically important changes and patient-acceptable symptom states with the anchor transition in function. Subgroups were identified for which independent thresholds should be defined. Patient-reported outcome measures were symptoms (pain, Oxford Hip Score [OHS], Hip disability and Osteoarthritis Outcome Score) and quality of life (European Quality of Life 5 Dimensions 3L questionnaire).

**Results:**

94.6% completed the anchor questions, of whom 80.1% reporting “much improved function” 1 year after surgery. Discriminative abilities of thresholds were not good (area under the curve < 0.8). Tercile-specific determination of thresholds improved discrimination and reliability (+10%). Minimal clinically important change values were higher for all outcomes (eg, change in OHS ≥ 24.5 vs ≥ 10.5) in patients with more severe preoperative symptoms. Patient-acceptable symptom state scores for European Quality of Life 5 Dimensions index (≥ 0.809) and OHS (≥ 40.5) showed good discrimination (area under the curve > 0.8). Patients with less symptoms required lower postoperative scores for reporting “much improved function” (postoperative OHS ≥ 38.5 vs 42.5). Tercile-specific thresholds did not improve accuracy of thresholds (Cohens kappa 42%).

**Conclusions:**

The present study demonstrates that patients with more severe preoperative symptoms require greater change scores to achieve clinically relevant improvements than patients with less severe preoperative symptoms. This study suggests that current one-size-fits-all thresholds for success of THA should be replaced with more nuanced thresholds.

**Level of evidence:**

Level III, Therapeutic Study.

## Introduction

Osteoarthritis of the hip is a debilitating disease and one of the leading causes of global disability [1]. Hip osteoarthritis affected 1.8 million patients in 2015 and is expected to affect 2.8 million in 2050 [2]. This corresponds to 6.6% of the total healthcare costs incurred for diseases of the musculoskeletal system and connective tissue and 0.5% of the total healthcare costs [3].

Nonoperative treatment consists of nonpharmacological interventions, such as education, weight loss, and physiotherapy, and pharmacological interventions (eg, nonsteroidal anti-inflammatory drugs or corticosteroids) [4,5]. When insufficient and advanced radiographic degenerative changes are evident, total hip arthroplasty (THA) is indicated.

In the past, treatment outcomes of THA for end-stage hip osteoarthritis have been mostly defined by implant survival, which suggest a successful treatment in > 95% of cases [[6], [7], [8]]. However, from a patient perspective, success is defined as satisfactory relief of symptoms, and by this measure, success rates of THA are only 80%-90% [[9], [10], [11]].

Predicting whether a treatment causes satisfactory relief of symptoms is pivotal in clinical decision-making. In clinical practice and research, symptoms are nowadays quantified with patient-reported outcome measures (PROMs) [12]. To allow interpretation of (changes in) PROMs scores, reference values with clinical meaning are required. As such, the most used thresholds are minimal clinically important change (MCIC) (for within-patient prepost comparison), patient-acceptable symptom state (PASS) (for postoperative score analysis) and minimal clinically important difference (MCID) (for between-patient comparison).

Determination of clinically relevant thresholds has been challenging. Published thresholds for PROMs carry significant variation and weaknesses. Thresholds differ significantly among studies and anchor questions (eg, satisfaction, transition in pain, transition in function), for example, Oxford Hip Scores (OHSs) from 16.5 [13] to 8 [10,14] and European Quality of Life Index scores from 0.33 to 0.41 [15]. These studies did not consider patient-specific variability, and those that did consider this variability have been based on small sample sizes (< 2000 patients), have been studied more than 10 years ago, are not fully available for change and postoperative scores and group differences, and vary in their anchor questions [[15], [16], [17], [18]].

Therefore, this study aims to define MCIC, PASS, and minimal MCID for all primary THAs, based on the Dutch Arthroplasty Registry data from 2016-2018. In extension of current knowledge, we assess whether these thresholds may differ for specific patient groups. We hypothesize that MCICs differ for patients of different terciles, based on their preoperative symptom severity scores.

## Material and methods

### Design

This is a retrospective registry study of all primary THAs in the Netherlands in the period 2016-2018 with a minimal follow-up of 1 year and completed preoperative and 12-month postoperative PROMs as registered in the Dutch Arthroplasty Registry. The MCIC and PASS for the postoperative score in each patient is determined for PROMs by receiver operating curve (ROC) analysis, using transition in function (score ≥ 6) as anchor. Using logistic regression, preoperative determinants of thresholds were identified. For significant determinants, independent ROC analyses were performed. Finally, we assessed the accuracy of the developed thresholds by comparing threshold-based interpretation of satisfaction with self-reported satisfaction.

### Data collection

The Dutch Arthroplasty Registry has a coverage of 100% of Dutch hospitals and the response rate for preoperative and 12-month postoperative PROMs over this period was 39% (33% in 2016, 39% in 2017, and 41% in 2018) [19]. Patient characteristics were sex, age, diagnosis, American Society of Anesthesiologists (ASA) classification, Charnley classification, body mass index (BMI), smoking status, and previous operations on the affected joint. Procedure characteristics were fixation (cemented vs uncemented), surgical approach, and used implant articulations. Collected PROMs were the numeric rating scales (NRSs) (0-10) for pain in rest and during activity, OHS (0-48), Hip disability and Osteoarthritis Outcome Score (HOOS) (0-100) to assess physical function, and the European Quality of Life 5 Dimensions 3L questionnaire to assess quality of life (QoL) (QoL index, −0.33 to 1.00) and self-reported health status (visual analog scale, 0-100). For the QoL index and OHS outcomes, low values indicate more severe symptoms, while for NRS pain scores and the HOOS, high values indicate more severe symptoms. Daily life functioning, defined using the anchor question, was inquired as “How did your general daily functioning change after the surgery of your hip?”, with response options as 1 “very much worse”, 2 “much worse”, 3 “a little worse”, 4 “unchanged”, 5 “a little better”, 6 “much better”, or 7 “very much better”. A successful THA was defined as a THA with a 12-month postoperative change in daily functioning score of 6 or more. All PROMs have been presented in the native language, in which they are validated [[20], [21], [22]].

### Statistical analyses

The statistical analysis, using SPSS V.27 (IBM SPSS Statistics, IBM Nederland B.V., The Netherlands), was performed as in previous studies with comparable objectives [11,15,16,18].

All analyses were performed and compared with the anchor question, defining a transition score ≥ 6 (“much better function”) as ‘successful’. Sensitivity analysis was performed using a more conservative anchor question score (transition score ≥ 5) [23]. Groups were compared using Mann-Whitney *U* tests due to non-normal distribution for continuous data, and Chi-square tests for categorical data. Within-group comparisons were performed using Wilcoxon matched-pair signed-rank tests.

ROC analyses were performed for all patients and PROMs (change over time and postoperative score) using transition in function (score ≥ 6) as anchor. Discriminative ability of obtained thresholds is quantified by the area under the ROC (area under the curve [AUC] < 0.8: fair, AUC > 0.8: good). Requirements for ROC analysis were assessed [24]. To choose the thresholds for ROC, the Youden method was used [13]. The 95% confidence intervals around the thresholds were estimated using percentile bootstrap methods, based on 10,000 random samples with replacement from the original data. Scores that best distinguish between self-reported success of surgeries were determined by ROC analyses. These thresholds with the higher area under the ROC describe the MCIC and PASS for the postoperative score in each patient.

Demographics, preoperative symptoms, surgical data, and PROMs are reported for successful vs unsuccessful surgeries. To test whether thresholds for success differed between demographic groups and patients with different baseline scores, logistic regressions were performed for patients with mild, mid, or severe preoperative symptoms; this categorization was performed for each PROM into even-numbered terciles. Transition in function after surgery was used as binary dependent outcome (anchor score ≥ 6). Baseline variables were demographic (age < 70 years vs ≥ 70 years, BMI < 30 kg/m^2^ vs ≥ 30 kg/m^2^, Charnley classification A vs B/C, and ASA classification I/II vs III/IV) and PROMs (as terciles, highest tercile: most severe symptoms). Operative variables (diagnosis, fixation, surgical approach, and used implants) were considered as covariates for PROMs in logistic regression. Thresholds were determined for each baseline group in independent ROC analyses if the interaction (score/change∗baseline variable) was significant after stepwise backwards elimination based on the likelihood ratio and *P* ≥ .05. Identified thresholds for each tercile were used to develop thresholds based on continuous preoperative data, using linear regression with baseline values as independent variable, and tercile-specific thresholds as dependent variable. Reliability and accuracy of these thresholds to predict self-reported change in daily functioning (anchor question) was assessed using Cronbach’s alpha-statistics (α < 0.5: unacceptable, α < 0.6: poor, α < 0.7: questionable, and α < 0.8: acceptable). MCIDs in preoperative symptom severity were determined, by assessing the difference in change in PROMs for a 1-point increase in self-reported change in daily functioning, adjusted for baseline tercile, using linear regression.

## Results

**Figure S1:**
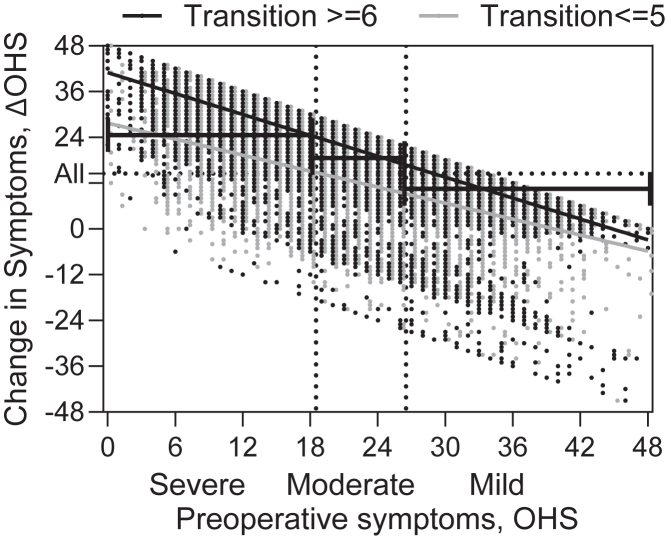
Transition scores by terciles of preoperative symptom severity of OHS. Graph of reported changes in OHS by preoperative symptom severity and terciles. OHS, Oxford Hip Score.

Between 2016 and 2018, 40,213 THAs with available PROMs data were registered (Table S1). Most patients were female; aged 70-79 years; overweight; classified as ASA class II; and Charnley class A, nonsmoking, and without history of previous intervention of the affected hip. Of the registered arthroplasties, the majority was indicated by osteoarthritis, performed uncemented, with a posterolateral approach, using ceramic-on-polyethylene articulations in THA. Of all registered THA patients with completed PROMs, 94.6% completed the anchor question, of whom 80.1% reporting “much improved function” or better (transition score ≥ 6), and 87.5% reporting “a little improved function” or better (transition score ≥ 5) (Fig. S1). Patients who reported successful surgeries were more frequently men, younger, leaner, healthier (based on ASA class), and less multilateral arthritic (based on Charnley class), as compared to patients who reported unsuccessful THAs (Table S1).

On average, THAs were highly effective in reducing osteoarthritis-specific symptoms and increasing quality of life (Table S2). Consistently across PROMs, groups with worse baseline scores had also greater improvements, yet postoperative scores remained ∼13%-37% lower than groups with better preoperative scores. In line, patients with worse baseline scores reported less frequently successful surgeries (∼6% less patients with transition score ≥ 6).

### MCIC and PASS scores

**Figure S2:**
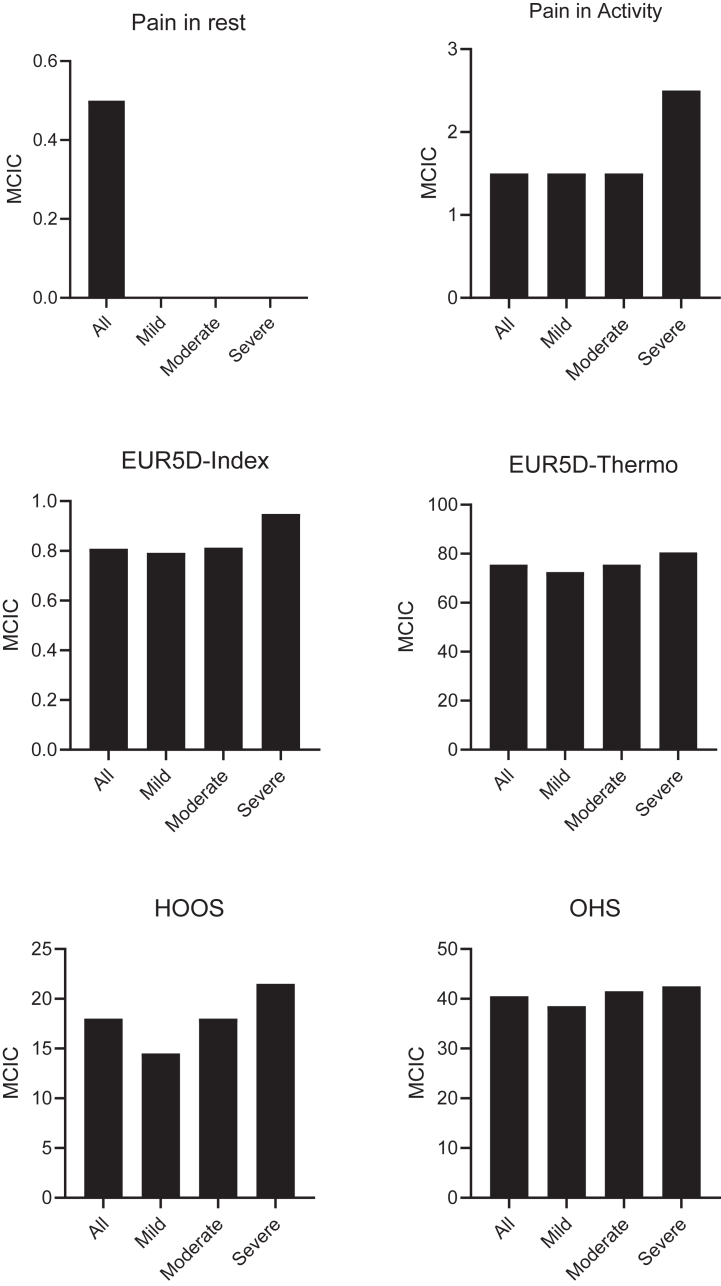
Changes in patient-reported outcome measures by transition status. Histogram of reported changes in scores are provided by transition status.

**Figure S3:**
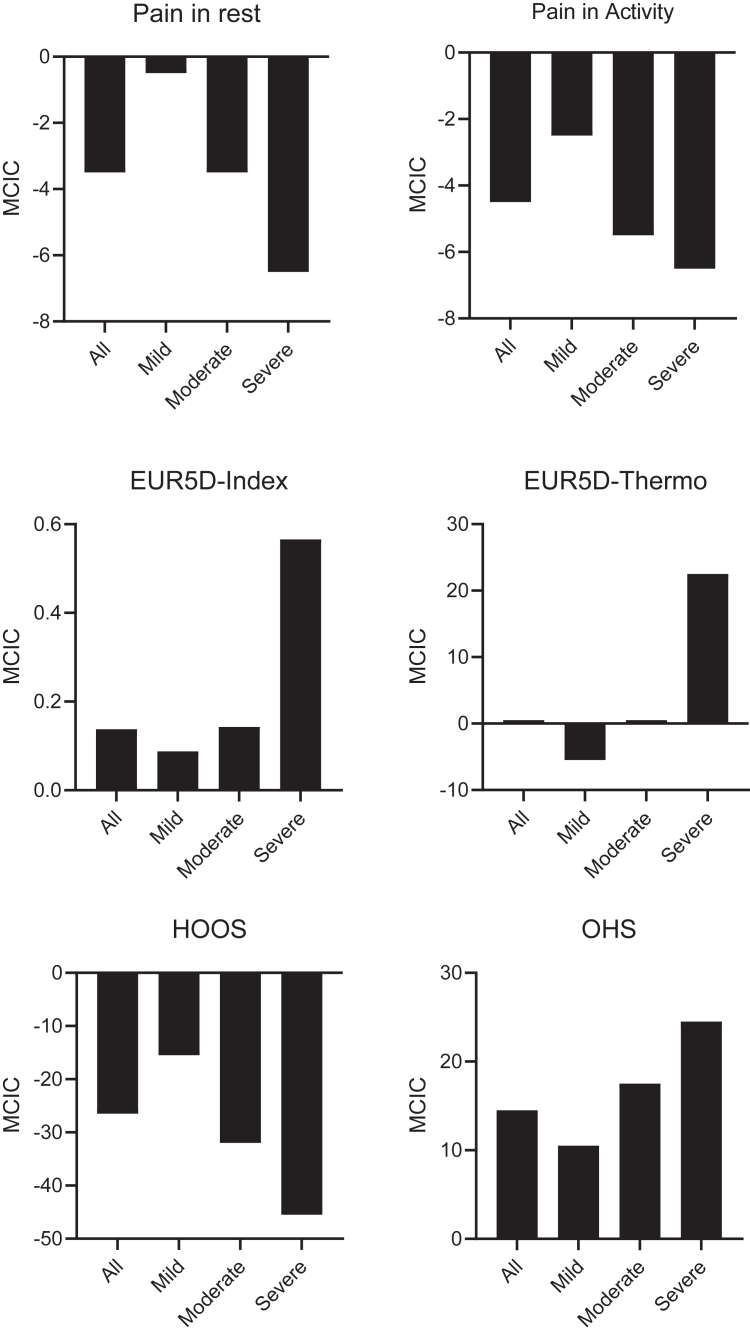
Postoperative patient-reported outcome measures by transition status. Histogram of reported postoperative scores are provided by transition status.

For all PROMs, postoperative and change scores are distinctly distributed between patients, who report successful surgeries vs patients, who report unsuccessful surgeries (Figure S1, Figure S2, Figure S3). For PASS, OHS ≥ 40.5 and QoL index ≥ 0.809 were good discriminative values (AUC > 0.8, Table S3). Correlations with anchor values are provided in Table S3. For MCIC, none of the investigated scores showed good discrimination.

### Determinants of thresholds and specific thresholds

For all outcome variables, except for pain in rest, thresholds differed by baseline tercile (Table S3, Figure S1, Figure S2). Consistently, patients with poorer preoperative scores reported larger changes in PROM scores that were associated with success. For example, the MCIC for OHS is 24.5 for patients with severe symptoms preoperatively, while patients with medium or mild symptoms have MCIC scores of 17.5 and 10.5, respectively. Discriminative abilities, that is, AUC, improved for tercile-specific analyses of MCIC scores. Best discrimination was consistently observed for patients with more severe preoperative symptoms. The discriminative ability of PASS scores by baseline tercile did not further improve discriminative ability of universal PASS scores.

Separate analysis by demographic variables, that were significant predictors in logistic regression, did not reveal relevant differences in MCIC and PASS scores (Table S1).

### Reliability of thresholds

The tercile method produced 10% more accurate estimates of self-reported success in surgery as compared to universal thresholds (Table S3). The continuous thresholds added another ∼3% increase to the reliability (Table S2). For the PASS scores, tercile-specific or continuous thresholds did not improve the accuracy of predicting satisfaction.

### MCID

The MCIDs in PROMs between 2 groups reporting a 1-score difference in transition are presented in Table S3. As for MCIC, MCIDs are sensitive to preoperative symptom severity.

## Discussion

In this study, we aimed to define accurate thresholds for PROMs after THA. For the first time, we discriminated patients by self-reported preoperative symptom severity, and observed significant differences for MCICs, the most reliable for the OHS. No significant differences were observed for PASS. Demographics were not relevant predictors of thresholds. This study also revealed significantly increased accuracy of tercile-specific thresholds. In clinical practice having better understanding of thresholds for PROMs after THA might be one of the factors to determine postoperative success and patient satisfaction. The more accurate thresholds suggest that patients with severe preoperative symptoms (eg, OHS 6) need at least a postoperative improvement of 18 points (postoperative OHS of 24) to be deemed satisfactory after THA (Fig. S1).

As hypothesized, obtained MCICs differed greatly by baseline terciles for all PROMs, and thus, patients with poorer preoperative scores (representing more severe symptoms) should be evaluated differently based on score changes compared to patients with better preoperative scores. For clinicians, it is important to have knowledge of how large the differences in MCICs are expected to be, and how they relate to the preoperative score. The difference in scores ranges from 30% of the entire scale for European Quality of Life Visual Analog Scale and HOOS up to 60% of the entire scale for NRS pain scores, QoL index, and OHS.

The identified thresholds in the present study for OHS (14.5, 10.5, 17.5, and 24.5 for all, mild, mid, and severe preoperative symptoms, respectively [Fig. S1]) are comparable or larger than thresholds published with comparable methodology. In a large UK cohort of 80,000 [10] and later upscaled to 300,000 patients [14], an OHS score of 7.5-9 was identified. The UK study was performed on a transition anchor inquiring “hip problems” after 6 months, while we inquired transition in function. Importantly, the UK study restricted their analysis to distinguishing patients who reported “a little improved” and those who reported “about the same” on transition, while we used the stricter “much improved” or better vs “a little improved” or worse, which explains the difference. Notably, the larger transition, as analyzed in the present study was found to be more relevant for patients [24]. An Austrian study (N = 3000) [13] reported comparable MCICs in a comparable analytic approach than we did in this study. In a 1995-2004 cohort of 800 UK THA patients [17], tercile-specific analyses were performed based on group-level differences instead of ROC analyses, with notably comparable results as the ones we obtained in our study, underscoring the validity of the present findings.

Rather expectedly, similar to previous findings on knee arthroplasty [18] and by Arden et al. [17] in 2011, PASS scores did not differ greatly between baseline terciles. The obtained PASS scores (42, 41.5, 40, and 37.5) in the present study are comparable to previous analyses [13,23,[25], [26], [27]] despite differences in study duration and anchor questions. Thus, for PASS, we conclude that tercile-specific requirements are not necessary for appropriate estimation of patient-perceived surgery success.

In both analyses, MCIC and PASS, demographics were no significant predictors of thresholds that associate with transition in function. There are some reports of sex and BMI differences for MCIC and PASS scores [15,17,18] but in those studies, preoperative symptoms were also different between these groups. Thus, demographic differences may be mediated by symptom severity and that tercile-specific analyses render demographic-specific analyses redundant.

Finally, we performed analyses of MCIDs between groups, by assessing the difference in change score between patients who reported transition in function different by 1 score. Comparable to the MCIC analyses, we observed differences in the MCID for baseline terciles. Patients with less severe self-reported preoperative symptoms had less differences in their clinically relevant change scores than patients with more severe symptoms, suggesting that patients with less severe symptoms are more sensitive in the perception of success for different changes in scores. Worth mentioning is that with less severe preoperative symptoms, there is automatically less room to improve postoperatively. This ensures that tercile-specific differences might not be clinically relevant.

This study presents 1 approach to define distinct thresholds by identifying these based on preoperative symptom severity, while other approaches exist too [14,28,29]. Comparative studies to evaluate the best valuation system have not yet been performed. In research, distinct or more nuanced thresholds offer a more accurate analysis of patient satisfaction by assessing proportions of patients achieving relevant improvements in addition to average satisfaction scores.

### Clinical implications

The determined thresholds can support clinicians in several ways. First, in the clinic, physicians and patients have a reference for a satisfactory result, when predicting postoperative improvement in symptoms after THA. We convincingly demonstrate that these reference values differ significantly for different patients (Fig. S1). For patients whose predicted outcome scores are less than these defined thresholds, THA is likely deemed unsatisfactory. THA can be reconsidered through shared decision-making with patients taking into account patient-specific (non)modifiable risk factors [30,31] and corresponding thresholds [32,33]. Second, clinical valuation and comparison is based on among others, the proportion of patients with satisfactory PROMs. Different studies have shown that average PROMs may differ between clinics or countries, and therefore, reference values for successful surgeries must account for discrepancies in average PROMs.

The present study shows that 1 approach defining distinct thresholds which are more sensitive to satisfactory results than one-size-fits-all thresholds. To illustrate, Figure S1 shows how the discriminative MCICs are better fitted between the adequate vs nonadequate transition group, as compared to the one-size-fits-all threshold (OHS, 14.5). For 1 in 10 patients (preoperative scores of > 34), the one-size-fits-all MCIC is not even achievable for patients because the scale ends at 48. In conclusion, this study suggests that replacement of universal thresholds with validated nuanced thresholds for PROMs might be one of the factors to predict success and patient satisfaction after THA.

### Strengths and limitations

The strengths of the present study are the large nationwide sample size of > 40,000 patients, the robust statistical analysis which is data-driven and validated.

The present study has limitations. Success of THA is defined by PROMs in patients without considering requirements for revision or surgeons’ assessment or definition of success. Data assessment performed by both the patient and physician might reveal a more objective and shared decided definition of success. Inherent to the patient-reported nature of PROMs, these can suffer from recall bias; ceiling effects; and time-consuming methodology, low completion rate, or transcription errors. In a recent study, Pronk et al. [34] evaluated that for THA, a minimally 60% of patients must complete PROMs to be adequately reflective of the entire population. In the present study, complete preoperative and 12-month postoperative data were obtained of 30% of patients registered in the Dutch Arthroplasty Registry database. This percentage is high in comparison to other studies, specifically given the population size of > 75,000 patients (THAs in the Netherlands 2016-2018), but results must therefore be interpreted with caution. Also, findings of this study must not necessarily be translatable to other countries, as previous international studies have shown [35,36]. Furthermore, recall bias might be present since correlations among postoperative scores, changes in scores, and patient satisfaction were not strong, and not all correlation coefficients were larger than 0.5. Both are statistical requirements for ROC analyses, and insufficient strengths of associations are also reflected by the poor discriminative ability expressed in AUC values and reliabilities. Notably, tercile-specific analyses provide better correlations than analyses of all patients combined. Finally, in this study, information that could also determine or influence perceived success of surgery, including patients’ expectations, socioeconomic status, physical health, and fitness, and information on rehabilitation program and adherence were not considered.

## Conclusions

The present study offers thresholds for patient-reported outcomes in THA by which patients have considered the surgery to be successful. Based on the discriminative ability of minimal clinically important differences, patients with more severe preoperative symptoms require greater change scores to achieve clinically relevant improvements than patients with less severe preoperative symptoms (eg, preoperative OHS of 6 needs to improve at least 18 points to be satisfied). These thresholds suggest current one-size-fits-all thresholds should be replaced with more nuanced thresholds. These nuanced thresholds help to better predict success rates after THA and might be one of the factors to support more specific counseling of patients with shared decision-making. Research efforts should continue to understand the variability in modifiable patient-specific risk factors and (nonmodifiable) comorbidities that currently limit prediction models.

## Conflicts of interest

The authors declare there are no conflicts of interest.

For full disclosure statements refer to https://doi.org/10.1016/j.artd.2025.101649.

## CRediT authorship contribution statement

**Julia E.J.W. Geilen:** Writing – review & editing, Writing – original draft, Investigation, Conceptualization. **Thomay-Claire A. Hoelen:** Writing – review & editing, Methodology, Investigation, Formal analysis, Data curation, Conceptualization. **Martijn G.M. Schotanus:** Writing – review & editing, Resources, Project administration, Methodology, Investigation, Formal analysis. **Wouter L. W. van Hemert:** Writing – review & editing, Validation, Methodology. **Anneke Spekenbrink-Spooren:** Writing – review & editing, Formal analysis, Data curation, Conceptualization. **Bert Boonen:** Writing – review & editing, Validation, Supervision, Formal analysis. **Jasper Most:** Writing – review & editing, Supervision.

## Figures and Tables

**Table S1:** Patient demographics and patient-reported success rates at 12 months after surgery.

Characteristic	All	Proportion successful	Chi-square, *P* value
All	40,213	79.1	
Sex, women	63.8	77.7	<.001
Sex, men	36.1	81.5	
Age			<.001
<50 years	3.9	83.5	
50-59 years	12.7	83.5	
60-69 years	34.5	82.7	
70-79 years	36.5	76.9	
80-89 years	12.3	69.5	
BMI			<.001
<18.5 kg/m^2^	0.6	74.7	
18.5-24.9 kg/m^2^	33.0	81.3	
25.0-29.9 kg/m^2^	43.1	79.6	
30.0-39.9 kg/m^2^	22.3	75.2	
≥40.0 kg/m^2^	1.1	75.3	
ASA classification			<.001
I	19.2	86.6	
II	64.5	78.8	
III, IV	16.4	71.1	
Charnley classification			<.001
A	44.9	79.9	
B1	30.2	79.0	
B2	20.7	78.6	
C	2.8	74.7	
Smoking, no	90.3	79.4	.002
Smoking, yes	9.7	77.2	
Previous surgery, no	96.2	79.3	<.001
Previous surgery, yes	3.8	73.0	
Side, left	46.0	79.3	.29
Side, right	54.0	78.9	
Diagnosis			<.001
Arthrosis	92.9	79.3	
Osteonecrosis	2.3	75.4	
Dysplasia	1.8	85.0	
Late post-traumatic	1.7	71.2	
Other diagnoses	1.3	*NA*	
Fixation			<001
Uncemented	69.5	81.5	
Cemented	21.8	72.5	
Hybrid: femur cemented	4.4	76.0	
Hybrid: acetabulum cemented	3.6	76.1	
Other/unknown	0.6	*NA*	
Surgical approach			<.001
Posterolateral	55.3	78.8	
Anterior	31.3	83.2	
Direct lateral	7.8	66.7	
Anterolateral	4.7	75.8	
Other	0.9	*NA*	

ASA, American Society of Anesthesiologists; BMI, body mass index; NA, not applicable.

Data are shown as % of all patients (column ‘all’) and as % patients within each row to report ‘much improved’ function 1 year after surgery. *P* values refer to the outcomes of Chi-square tests for testing whether demographics differed between patients reporting successful surgeries vs unsuccessful surgeries. 2185 patients (5.4%) did not provide data on success, that is, transition in function.

**Table S2:** Patient-reported outcome measures before and 1 year after primary total hip arthroplasty.

PROM	N	Preoperatively			12-month postoperatively			Delta			Patients, “much improved”, %
		Mean	SD	Median	Mean	SD	Median	Mean	SD	Median	
Pain, rest, NRS											
All	39,304	7.2	2.1	8	1.5	2.2	0	−5.7	2.8	−6	
Poor (≥9)	9949	9.3	0.5	9	1.8	2.5	1	−7.5	2.5	−9	77
Mid (7-8)	18,522	7.6	0.5	8	1.5	2.2	0	−6.1	2.2	−7	79
Good (≤6)	10,833	4.4	1.7	5	1.1	1.8	0	−3.3	2.4	−4	82
Pain, activity, NRS											
All	39,268	5.2	2.6	5	0.9	1.8	0	−4.3	2.8	−5	
Poor (≥8)	7966	8.4	0.7	8	1.3	2.2	0	−7.1	2.3	−8	78
Mid (4-7)	20,310	5.7	1.1	6	0.9	1.8	0	−4.7	2.0	−5	80
Good (≤3)	10,992	1.8	1.1	2	0.5	1.3	0	−1.3	1.6	−2	80
Quality of life, QoL index											
All	39,603	0.566	0.270	0.693	0.862	0.184	0.893	0.295	0.284	0.225	
Poor (<0.569)	12,430	0.196	0.137	0.216	0.806	0.226	0.843	0.610	0.241	0.645	76
Mid (0.569-0.775)	22,330	0.712	0.069	0.693	0.879	0.161	0.897	0.166	0.164	0.225	80
Good (>0.775)	4843	0.837	0.051	0.807	0.926	0.120	1.000	0.089	0.127	0.139	83
Health status, QoL-VAS											
All	39,612	66	20	70	76	21	80	10	26	10	
Poor (≤59)	12,189	41	15	48	70	23	75	29	27	30	74
Mid (60-77)	14,349	68	5	70	76	19	80	7	20	10	79
Good (≥78)	13,074	86	6	85	82	20	87	−4	20	0	83
HOOS											
All	37,495	49	18	46	13	15	9	−35	20	−35	
Poor (≥57)	10,002	71	10	68	18	17	13	−53	19	−55	76
Mid (42-56)	15,396	49	5	51	13	14	9	−36	14	−38	81
Good (≤41)	12,097	30	8	30	10	12	5	−19	14	−22	81
Oxford Hip Score											
All	36,353	23	9	24	41	9	44	18	11	19	
Poor (≤18)	10,991	13	4	13	39	9	42	27	9	29	76
Mid (19-26)	13,461	23	3	23	41	8	44	18	8	21	80
Good (≥27)	11,901	33	4	32	43	9	46	10	9	12	81

HOOS, hip disability and osteoarthritis outcome score; NRS, numeric rating scale; QoL-VAS, self-reported health status, by visual analog scale; SD, standard deviation.

All changes are statistically significant (*P* < .001, Mann-Whitney *U* test).

**Table S3:** Thresholds for transition (score ≥ 6) by baseline terciles.

PROM	Subgroup	MCIC		PASS	
		Threshold	AUC, standard error	Threshold	AUC, standard error
Pain, rest, 0-10	All	≤−3.5	0.633, 0.004	≤0.5	0.709, 0.004
Pain, rest, 0-10	≤3	≤−0.5	0.629, 0.008		
Pain, rest, 0-10	4-7	≤−3.5	0.700, 0.005		
Pain, rest, 0-10	≥8	≤−6.5	0.732, 0.008		
Pain, activity, 0-10	All	≤−4.5	0.714, 0.004	≤1.5	0.749, 0.004
Pain, activity, 0-10	≤6	≤−2.5	0.691, 0.007	≤1.5	0.721, 0.007
Pain, activity, 0-10	7-8	≤−5.5	0.756, 0.005	≤1.5	0.760, 0.005
Pain, activity, 0-10	≥9	≤−6.5	0.757, 0.007	≤2.5	0.762, 0.007
QoL index, −0.329-1	All	≥0.138	0.667, 0.004	≥0.809	0.805, 0.003
QoL index, −0.329-1	< 0.569	≥0.566	0.759, 0.005	≥0.792	0.814, 0.005
QoL index, −0.329-1	0.569-0.775	≥0.143	0.785, 0.004	≥0.813	0.807, 0.004
QoL index, −0.329-1	> 0.775	≥0.088	0.754, 0.010	≥0.949	0.757, 0.011
QoL-VAS, 0-100	All	≥0.5	0.640, 0.004	≥75.5	0.742, 0.003
QoL-VAS, 0-100	≤59	≥22.5	0.705, 0.006	≥72.5	0.753, 0.005
QoL-VAS, 0-100	60-77	≥0.5	0.737, 0.005	≥75.5	0.747, 0.005
QoL-VAS, 0-100	≥78	≥−5.5	0.696, 0.006	≥80.5	0.709, 0.006
HOOS, 0-100	All	≤−26.5	0.707, 0.004	≤18	0.789, 0.003
HOOS, 0-100	≤41	≤−15.5	0.755, 0.006	≤14.5	0.768, 0.006
HOOS, 0-100	42-56	≤−32	0.795, 0.005	≤18	0.808, 0.005
HOOS, 0-100	≥57	≤−45.5	0.767, 0.007	≤21.5	0.797, 0.006
OHS, 0-48	All	≥14.5	0.711, 0.004	≥40.5	0.806, 0.003
OHS, 0-48	≤18	≥24.5	0.805, 0.006	≥38.5	0.833, 0.005
OHS, 0-48	19-26	≥17.5	0.804, 0.005	≥41.5	0.820, 0.005
OHS, 0-48	≥27	≥10.5	0.754, 0.006	≥42.5	0.778, 0.006

AUC, area under the curve; HOOS, hip disability and osteoarthritis outcome score; MCIC, minimal clinically important change, NRS, numeric rating scale; OHS, Oxford Hip Score; PASS, patient-acceptable symptom state; QoL, quality of life; QoL-VAS, self-reported health status, by visual analog scale.

Thresholds were determined by the Youden method.

## Appendix A Supplementary data

Conflict of Interest Statement for Geilen

Conflict of Interest Statement for Hoelen

Conflict of Interest Statement for Schotanus

Conflict of Interest Statement for van Hemert

Conflict of Interest Statement for Spekenbrink-Spooren

Conflict of Interest Statement for Boonen

Conflict of Interest Statement for Most
