# Supplementary material for: Defining Clinically Meaningful Thresholds for 12-Month Patient-Reported Outcomes in Total Hip Arthroplasty; Toward Improving Threshold Accuracy
Source: Arthroplast Today. 2025 Mar 3;32:101649. doi: 10.1016/j.artd.2025.101649 (PMC11926718; doi:10.1016/j.artd.2025.101649)
Supplement: Conflict of Interest Statement for Spekenbrink-Spooren [file mmc5.docx]

# CONFLICT OF INTEREST STATEMENT

***American Association of Hip and Knee Surgeons***

(Adopted from the American Academy of Orthopaedic Surgeons disclosure statement)

The following form **must be filled out completely and submitted by each author (example, 6 authors, 6 forms).**

**All items require a response. If there is no relevant disclosure for a given item, enter "*None*.”**

***Defining clinically meaningful thresholds for 12-month patient-reported outcomes in total hip arthroplasty; towards improving threshold accuracy***

1. Royalties from a company or supplier (The following conflicts were disclosed) ***None***

2. Speakers bureau/paid presentations for a company or supplier (The following conflicts were disclosed) ***None***

3A. Paid employee for a company or supplier (The following conflicts were disclosed) ***None***

3B. Paid consultant for a company or supplier (The following conflicts were disclosed) ***None***

3C. Unpaid consultants for a company or supplier (The following conflicts were disclosed) ***None***

4. Stock or stock options in a company or supplier (The following conflicts were disclosed) ***None***

5. Research support from a company or supplier as a Principal Investigator (The following conflicts were disclosed) ***None***

6. Other financial or material support from a company or supplier (The following conflicts were disclosed) ***None***

7. Royalties, financial or material support from publishers (The following conflicts were disclosed) ***None***

8. Medical/Orthopaedic publications editorial/governing board (The following conflicts were disclosed) ***None***

9. Board member/committee appointments for a society (The following conflicts were disclosed) ***None***

Author Name (Print or Type) Author Signature Date

Anneke Spekenbrink-Spooren AS 13 September 2024
